# Impact of agricultural land use rights transfer on carbon emission intensity of cultivated land--Empirical evidence based on panel data of 30 provinces in China

**DOI:** 10.1371/journal.pone.0322714

**Published:** 2025-05-02

**Authors:** Yuetang Chen, Haofan Zhang

**Affiliations:** School of Public Administration and Law, Hunan Agricultural University, Changsha City, Hunan Province, China; Directorate of Rapeseed-Mustard Research, INDIA

## Abstract

In the context of high-quality agricultural development, farmers increasingly engage in agricultural land use rights transfer(ALURT) to achieve large-scale operations and improve agricultural production efficiency. However, large-scale agricultural operations often lead to mechanized production, which may cause higher carbon emissions, contradicting the principles of green agricultural development. This study aimed to assess the actual impact of ALURT on the carbon emission intensity (CEI) of croplands and explore the role of agricultural large-scale operations in this relationship. To achieve this, the CEI of arable land across 30 provinces in China from 2014 to 2022 was measured, and the impact of ALURT on the CEI was analyzed using a two-way fixed-effects model, a mediated-effects model, and a threshold-effects model. These findings suggested that the total carbon emissions and CEI of arable land in China have declined annually since 2015. Southeastern coastal provinces, including Shanghai and Zhejiang, have the highest CEI of croplands. ALURT significantly reduced the CEI for arable land. Moreover, mechanism testing revealed that large-scale operations did not have a mediating effect but instead exhibited a threshold effect. When the scale of agricultural operations grew to the threshold, the inhibition of ALURT on CEI could be amplified.

## Introduction

As global climate change intensifies, reducing greenhouse gas (GHG) emissions has become a significant challenge for the international community. Agriculture, a fundamental sector of the national economy, is a significant contributor to GHG emissions. The global food system accounts for more than one-third of anthropogenic GHG emissions, based on a 2021 report by the Food and Agriculture Organization of the United Nations. Therefore, thoroughly investigating the factors influencing agricultural carbon emissions and identifying reduction pathways are vital for achieving global carbon reduction targets and promoting sustainable agricultural development. The reduction of carbon emissions from agriculture has garnered significant academic attention, leading to a wealth of valuable research findings.

ALURT has served as a vital reform measure in China in recent years, profoundly affecting agricultural production methods, resource allocation, and land-use efficiency[1]. This policy aims to enhance agricultural production efficiency and boost the rural economy by promoting redistribution of land resources and encouraging large-scale and intensive agricultural operations. However, this may also lead to environmental issues, particularly variations in the CEI of the arable land. This study systematically investigated the impact of ALURT on cropland CEI by collecting and analyzing panel data from 30 provinces in China. Specifically, a double fixed-effect model and an instrumental variable model (IVM) were established to thoroughly analyze the relationship between ALURT and cropland CEI based on the characteristics of the research questions and data. Additionally, we considered the moderating effect of the scale of agricultural operations on the relationship between ALURT and cropland CEI. This comprehensive analysis aimed to reveal the mechanism where the impact of ALURT on cropland CEI. This study found that ALURT had an inhibitory effect on cropland CEI, which is consistent with the conclusions of previous research [2,3], However, the scale of operation did not mediate the relationship between ALURT and cropland CEI; instead, it exhibited a threshold effect..

It is important to clarify that in existing studies, agricultural CEI is typically calculated by dividing carbon emissions from agricultural activities by the value of agricultural output [4,5]. However, some studies have used alternative approaches such as dividing carbon emissions by agricultural output [6] or cultivated land areas [7]. This study argues that the value of agricultural output is an economic metric affected by various factors, including market demand, price fluctuations, and policy adjustments, making it inherently volatile and uncertain. Similarly, crop yields are affected by non-agricultural factors such as climate and soil conditions, in addition to planting techniques and management practices. Therefore, these two calculation methods can lead to significant variations in CEI estimates across years or regions, thereby reducing data comparability and stability. In contrast, calculating the CEI by dividing agricultural carbon emissions by cultivated land area provides a more direct reflection of the impact of agricultural production activities on carbon emissions. This approach also eliminates the influence of external economic and climatic factors, which enhances the comparability of CEI across different regions and soil types. Therefore, this study considered the first two calculation methods inappropriate and adopted a cultivated land area-based approach to determine the carbon emission intensity of cultivated land. Moreover, this study offers a practical pathway for other developing countries to achieve low-carbon, sustainable agricultural development by optimizing land resource allocation and improving agricultural production efficiency.

## Literature review

Research on carbon emissions from croplands has primarily focused on the measurement of carbon emissions from regional cropland utilization, spatiotemporal features of these emissions, and their controlling factors.

According to the plant life cycle, cropland carbon sources primarily include methane emitted by crops during growth [8], fertilizers, pesticides, agricultural films used in cultivation, agricultural machinery, and carbon emissions from tilling, irrigation, and straw burning [9]. Carbon emissions from croplands are commonly measured by multiplying the amount of each carbon source consumed by its corresponding emission factor [10]. This study adopted this method to calculate carbon emissions from croplands.

The study of the spatiotemporal features of carbon emissions and the controlling factors has indicated that the evolution of carbon emissions varies across different areas owing to the differences in climate, topography, cultivation practices, and dominant industries. Most studies on the spatiotemporal features of agricultural GHG emissions in China indicate an increasing-decreasing trend [11]. However, other studies have identified a stepwise upward trend in carbon emissions over time, with a gradually slowing growth rate [12] and an expanding area of high carbon emissions [13]. Factors affecting carbon emissions from croplands include the adoption of agricultural conservation practices, biochar application, agricultural industry structure, environmental regulation, urbanization, climate, and fertilizer application intensity. The degree and direction of each factor’s influence on GHG emissions vary significantly across regions [14–19].

Against the significant rural labor outflow, numerous farmers choose to outsource the management rights of their arable land [20]. The key variables influencing the decisions of farmers to transfer their farmland use rights include non-farm employment, household income, topographical characteristics, farm size, agricultural productivity, socioeconomic factors, rural socioeconomic structure, level of rural governance, and agricultural socialization services [20–23]. The ALURT not only increases farmers’ income [24] but also affects the green transformation of agriculture [25] and promotes sustainable agricultural development [26]. As China’s urbanization has accelerated and the demand for agricultural modernization has increased, research has shifted towards deeper institutional innovations, the exploration of transfer modes, and achieving efficient land resource use while ensuring food security and ecological balance. This study examined the relationship between cropland CEI and ALURT in China (excluding Tibet because of its unique agricultural characteristics). The objective was to provide a scientific basis for proposing policies that promote low-carbon agricultural development.

## Theoretical analysis and research hypotheses

### Ability of ALURT to reduce cropland CEI

The positive impact of ALURT in reducing the CEI of arable land primarily results from the facilitation of optimal land resource allocation and intensive agricultural production. Large-scale management enhances agricultural mechanization [27], improves operational efficiency, and potentially reduces energy consumption and carbon emissions per unit area. Furthermore, the transferred land is more likely to adopt soil management practices, such as crop rotation, fallow periods, and straw return, which enhance the carbon sink function of the soil [28] and indirectly reduce atmospheric carbon levels. ALURT also aids in optimizing the agricultural structure, and upgrading the agricultural industrial structure can lead to reduced carbon emissions [17]. Underdevelopment of the land use rights transfer market may lead to land abandonment [29]. Therefore, facilitating smooth ALURT can prevent land abandonment, improve land use efficiency, and reduce carbon emissions associated with ineffective land use.

Hypothesis 1: ALURT can decrease the CEI of arable land.

### The mechanisms of ALURT’s impact on the CEI of croplands

The scale of agricultural operations plays an important role in the effect of ALURT on cropland carbon emissions. When the scale of operation is relatively small, farmers face challenges in adopting high-efficiency emission reduction measures owing to limitations in resources and technology [30]. As farmland is transferred, the scale of farmland operations expands, and mechanization and advanced agricultural management practices associated with large-scale operations can significantly reduce carbon emissions per unit area of cropland [28]. This outcome arises from the optimal allocation of resources due to economies of scale [31], which enhances overall agricultural production efficiency. Therefore, this study argues that large-scale operations mediate the relationship between ALURT and CEI of cropland.

In summary, Hypothesis 2 was proposed as follows: Large-scale operations serve as intermediaries in the effect of ALURT on cropland CEI.

**Fig 1:**
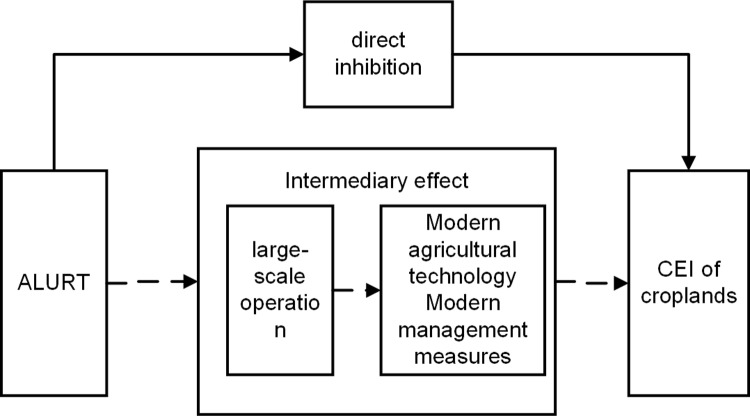
A theoretical framework for analyzing the impact of ALURT on CEI of croplands. Solid arrows denote direct causal pathways, while dashed arrows represent mediated relationships within the conceptual framework.

According to the theoretical analysis, a theoretical framework was developed to analyze the effect of ALURT on arable land CEI (Fig 1).

## Methods and materials

### Research methodology

#### Two-way fixed-effects model.

This study adopted panel data and a two-way fixed-effects model, which is a common selection for such data. The two-way fixed-effects model accounts for both individual and time fixed effects, thereby controlling for the impact of individual and temporal characteristics on the dependent variable. This approach enhances the focus on the causal relationship between dependent and explanatory variables. The equation is as follows:


$$CLCEI_{it}=\alpha +\beta ALT_{it}+\delta X_{\text{it}}+\varepsilon_{i}+\gamma_{t}+\mu_{it}$$ (1)

where for the province i in year t, CLCEI_it_ represents the cultivated land CEI; ALT_it_ is the level of ALURT; X_it_ denotes the control variable; ε_i_ represents the spatial effect; γ_t_ denotes the temporal effect; µ _it_ is the random error term, and ɑ, β, and δ are the estimated parameters.

#### Instrumental variable model (IVM).

To avoid the estimation bias caused by endogeneity, this study adopted the carefully selected instrumental variable, “the proportion of migrant laborers”. This variable was strongly correlated with the decision to transfer agricultural land but was not directly related to the model’s error term or the dependent variable of carbon emissions from cultivated land. The IVM effectively isolates the true effect of ALURT while preserving the explanatory power of the model, thereby enhancing the exogeneity and credibility of the estimation results. The specific equation is as follows:


$$\overset{―}{ALT_{\text{it}}}=\alpha_{1}+\beta_{1}IV_{\text{it}}+\sum \delta X_{\text{it}}+\varepsilon_{\text{i}}+\gamma_{\text{t}}+\mu_{\text{it}}$$ (2)

where $\overset{―}{\text{AL}{\text{T}}_{\text{it}}}$ is the level of ALURT with instrumental variable fitting; β_1_ represents the coefficient of the instrumental variable; α_1_ represents the intercept term; and IV_it_ represents the instrumental variable, with the remaining variables exhibiting the same meaning as in Equation (1). In the subsequent IVM process, the fitted level of ALURT was incorporated into Equation (1) using the formula provided in Equation (3):


$$CLCEI_{\text{it}}=\alpha_{3}+\beta_{3}\overset{―}{ALT_{\text{it}}}+\sum \delta X_{\text{it}}+\varepsilon_{\text{i}}+\gamma_{\text{t}}+\mu_{\text{it}}$$ (3)

where α_3_ is the intercept term, and β_3_ is the coefficient of ALURT with instrumental variable fitting. At this stage, bias in β_3_ due to endogeneity can be excluded.

#### Mediation effects model.

In this study, a path analysis was employed to test whether large-scale arable land operations had a mediating effect. The analysis follows a three-step approach. First, the direct effect of the independent variable on the dependent variable is estimated. Second, whether the independent variable has a significant effect on the mediator variable is assessed. Third, the joint effect of the independent variable and the mediator variable on the dependent variable is assessed to be significant or not. By analyzing the results of each stage, the presence of a mediating effect can be determined. The formulas for these three stages are presented in Equations (4)–(6):


$$Y=\text{c}X+e_{1}$$ (4)

$$M=\text{a}X+e_{2}$$ (5)

$$Y=c^{\prime }X+bM+e_{3}$$ (6)

where X denotes the independent variable, Y denotes the dependent variable, M denotes the mediator variable, e denotes the error term for each model, and c, a, c’, and b are the impact coefficients. If the coefficients c, a, c’, and b in Equations (4)–(6) are all statistically significant and c’ < c, this indicates a partial mediation effect, where c = ab + c’. If only c’ is not significant, this suggests a full mediation effect. However, if the regression results of Equation (5) or Equation (6) are not significant, it implies that the variable does not act as a mediator.

#### Threshold effect model.

To examine the intrinsic mechanism through which the level of ALURT could influence the CEI from croplands, ALURT may directly influence agricultural scale operations, thereby mitigating carbon emissions. Consequently, the following threshold effect model was established:


$$CLCEI_{it}=\phi_{0}+\phi_{1}ALT\text{it}\times \text{I(}LLO\text{it}\leq \theta )+\phi_{2}ALT_{\text{it}}\times I(LLO_{\text{it}}>\theta )+\phi 3X_{\text{it}}+\varepsilon_{\text{i}}+\mu_{\text{it}}$$ (7)

where LLO is the threshold variable indicating the level of large-scale operation, and I (·) represents the function that returns a value of 1 when meeting the conditions in parentheses. Instead, it returns a value of 0. Equation (4) represents the single-threshold model, which can be extended to a multi-threshold model based on the results of the subsequent tests.

#### Measurement of cropland CEI.

The adopted dependent variable was cropland CEI, which is defined as the ratio of carbon emissions from croplands to cropland areas. Carbon emissions were assessed using the carbon emission factor method proposed by IPCC. The IPCC itself does not provide a single standardized carbon emission factor applicable to all types of agricultural activities or inputs. Instead, the IPCC’s Good Practice Guidance and Uncertainty Management in National Greenhouse Gas Inventories states that “country-specific emission factors should be used wherever possible to reflect a country’s specific conditions and agricultural practices”. Accordingly, this study employed the carbon emission factors commonly used by Chinese researchers (the specific sources and coefficients are presented in Table 1). The carbon emission coefficient for agricultural irrigation was 25 kg/hm^2^. In later calculations, referring to the practice of Li Bo et al[32], the actual value of the coefficient of agricultural irrigation, considering the coefficient of thermal power, was 20.476 kg/hm^2^. Following existing studies [32,33], this study measured carbon emissions from six sources: tillage, irrigation, diesel fuel, fertilizer, pesticide, and agricultural film. The calculation formula is as follows:

**Table 1:** Carbon emission coefficients of major carbon sources utilized by arable land.

Carbon source	Carbon emission coefficient	Data source
tillage	3.126 kg·hm^-2^	College of Agronomy and Biotechnology, China Agricultural University [34]
irrigation	25 kg·Cha^-1^	Dubey [35]
agricultural diesel	0.5927 kg·kg^-1^	IPCC [32]
chemical fertilizer	0.8956 kg·kg^-1^	T.O.west [36]
pesticide	4.9341 kg·kg^-1^	Oak Ridge National Laboratory, USA [37]
agricultural film	5.18 kg·kg^-1^	Institute of Agricultural Resources and Ecological Environment, Nanjing Agricultural University [32]


$$CLCE=\sum CE_{i}=\sum Q_{i}Y_{i}$$ (8)

where CLCE indicates carbon emissions from cropland; CE_i_ represents the carbon emissions generated by carbon sources such as tillage, irrigation, agricultural diesel, agricultural film, pesticides, and chemical fertilizers in the farming process; Q_i_ represents the amount of each carbon source; and Y_i_ represents the corresponding carbon emission coefficients of each carbon source.

### Data sources

After 2002, China entered the rapid development phase for ALURT. The State Council proposed a Decision on Deepening Reforms and Strict Land Management in 2004, permitting the legal transfer of construction land rights to villages, market towns, and established towns. In 2014, the General Office of the State Council and the General Office of the Central Committee of the Communist Party of China (CPC) jointly proposed Opinions on Guiding the Orderly Transfer of the Right to Operate on Rural Land and Developing Agricultural Adaptive Scale Operations, which mandated regions to promote ALURT and develop moderate-scale operations. Therefore, this study began in 2014. Owing to the unique agricultural characteristics and lack of data for Tibet, this study was limited to 30 provinces (including autonomous regions and municipalities directly under the central government) in mainland China without Tibet.

Table 2 presents the definitions and values of the variables. Data on agricultural diesel fuel, chemical fertilizers, pesticides, agricultural films, total sown area of crops, and total power of agricultural machinery were mainly provided by the China Rural Statistics Yearbook. The data on the area of family contracted arable land, the total area of transferred family contracted arable land, the number of aggregated rural laborers, and the number of laborers going out to work were provided by the China Rural Business Management Statistical Yearbook. Regional public fiscal allocations, including expenditures on water resources management, forestry, and agriculture; investments in environmental protection and energy conservation; the extent of cultivated land; irrigated land area; employment figures in the primary sector; and the area of crops impacted by natural disasters are drawn from the China Statistical Yearbook. The missing values were supplemented using linear interpolation. To prevent bias from extreme values, all data were trimmed bilaterally during the analysis.

**Table 2:** Definitions of variables and descriptive statistics.

Variable classification	variable name	Variable Meaning and Assignment	average value	standard deviation	minimum value	maximum values
Dependent variable	CEI from croplands	Carbon emissions from cropland utilization/cropland area	0.053	0.028	0.014	0.142
Core explanatory variables	Level of ALURT	Total area of family-contracted arable land transferred/area of arable land operated by family contractors	0.384	0.340	0.041	5.235
Instrumental variable	Percentage of labor force working outside the home	Number of outworkers in the labor force/aggregated labor force	0.417	0.093	0.082	0.658
Intermediary Variables	Level of large-scale operation	Total sown area of crops/number of persons employed in the primary sector	12.764	6.606	4.896	41.571
Control variable	Input intensity of agricultural machinery	Total power of agricultural machinery/total sown area of crops	0.435	0.148	0.211	0.890
	Level of financial support for agriculture	Expenditures on agriculture, forestry and water affairs/local general public budget expenditures	0.115	0.035	0.040	0.204
	index of replanting	Total sown area of crops/area of arable land	1.340	0.426	0.496	2.389
	Agricultural disaster rate	Area of crops affected by the disaster/total sown area of crops	0.122	0.106	0.004	0.696
	environmental regulation	Expenditures on environmental protection and energy conservation/local general public budget expenditures	0.029	0.010	0.011	0.068

## Results

### Measuring agricultural CEI in China

**Fig 2:**
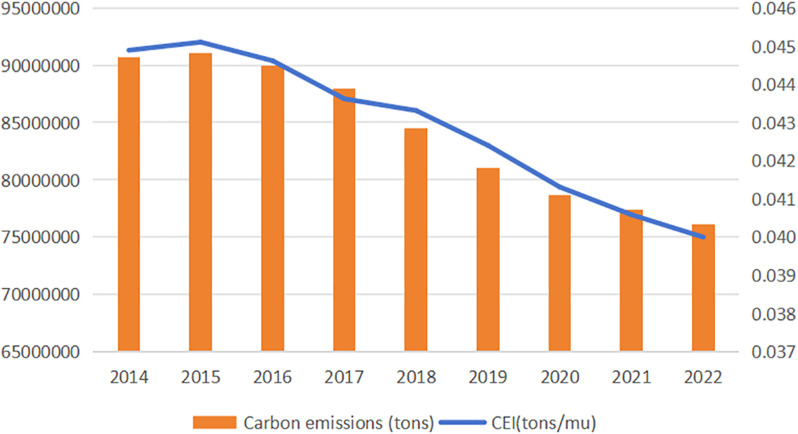
Temporal Dynamics of Cropland Carbon Metrics in China: Aggregate Emissions and Intensity Trends (2014–2022). (a) The orange bar charts delineate interannual variations in aggregate carbon emissions; (b) The blue line graph characterize the non-linear trajectory of CEI, exhibiting an initial increase in the first observational year followed by a progressive decline in subsequent years.

According to the index settings, the CEI and total carbon emissions of cultivated land across the 30 provinces from 2014 to 2022 were calculated, with the trends depicted in Fig 2. The data indicated that China’s CEI and total carbon emissions peaked in 2015 at 0.045 tons/mu(Mu is a proprietary Chinese unit of measurement of land area, where 1 mu = 0.0667 hectares) and 91,034,190 tons, respectively. Between 2015 and 2022, both the total carbon emissions and CEI declined annually, with the total emissions decreasing from 91,034,190 tons in 2015–76,098,890 tons in 2022 and the CEI falling from 0.045 tons/mu in 2015 to 0.040 tons/mu in 2022.

**Fig 3:**
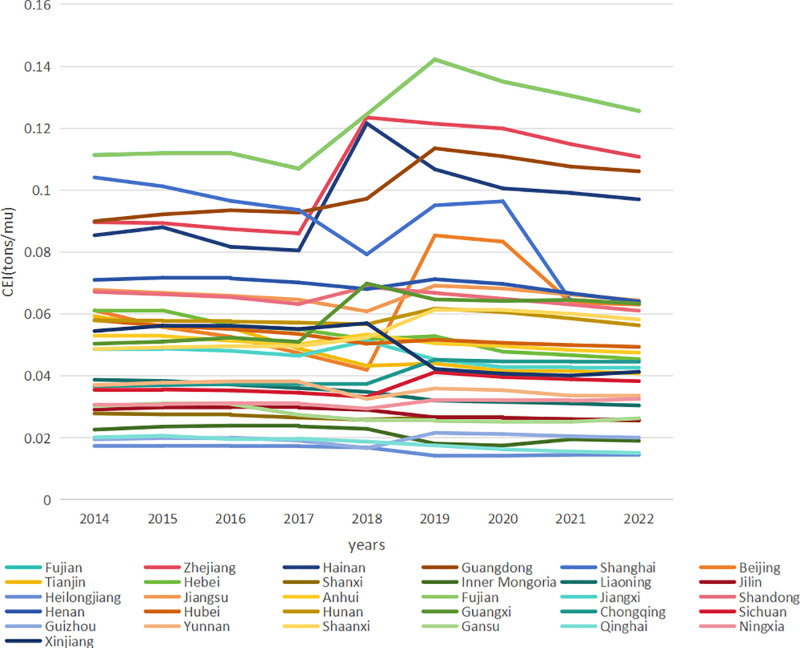
Line graph of annual changes in CEI for cropland in different provinces. (a) The X-axis represents the year (2014-2022); (b) The Y-axis represents the CEI in (tons/mu); (c) The line graphs of different colors represent the annual changes of CEI in different provinces.

The distribution of the CEI by province for each year is shown in Fig 3. Between 2014 and 2022, the CEI values in most provinces fluctuated within a narrow range, exhibiting no obvious upward or downward trend. Notably, four provinces (Fujian, Zhejiang, Hainan, and Guangdong) had the highest CEI values. These southeastern coastal regions, characterized by dense populations and limited arable land, tend to employ intensive high-input agricultural production models to maximize the yield per unit area. Such practices often involve the substantial use of agricultural films, pesticides, and chemical fertilizers, which directly or indirectly contribute to higher carbon emissions per unit. The CEI values in Fujian, Zhejiang, Hainan, and Guangdong provinces also showed significant fluctuations, with a trough in 2017, followed by a period of rapid growth lasting 1–2 years and a gradual decline. This pattern aligns with the global trends in carbon emissions. In March 2019, the International Energy Agency (IEA) released its report on “State of the World’s Energy and Carbon Dioxide 2018”, highlighting that “global energy demand grew strongly in 2018, and carbon emissions hit a record high”. This surge can be attributed to the accelerated modernization of agriculture and intensification of economic activities in these regions, leading to increased carbon emissions from energy consumption and fertilizer use in the agricultural sector. In contrast, other regions displayed a more subdued response to global carbon emission trends. This can be attributed to lagging agricultural development, lower economic activity intensity, and limited implementation of agricultural carbon emission management policies.

### Influence of ALURT on the CEI of arable land

#### Multicollinearity analysis.

Multicollinearity analysis(the results are shown in Table 3) of the explanatory variables in the model revealed that all variables had tolerance values greater than 0.1 and variance inflation factors (VIF) less than 10. These results indicated an absence of multicollinearity among the variables, providing a crucial prerequisite for ensuring the validity, stability, and reliability of the model.

**Table 3:** Multicollinearity test results for variables.

Variable	VIF	1/VIF
Level of financial support for agriculture	1.76	0.568676
Index of replanting	1.45	0.688974
Level of ALURT	1.41	0.711642
Agricultural disaster rate	1.31	0.761559
Input intensity of agricultural machinery	1.20	0.835747
Environmental regulation	1.14	0.873363
Mean VIF	1.38	

#### Main effects analysis.

**Fig 4:**
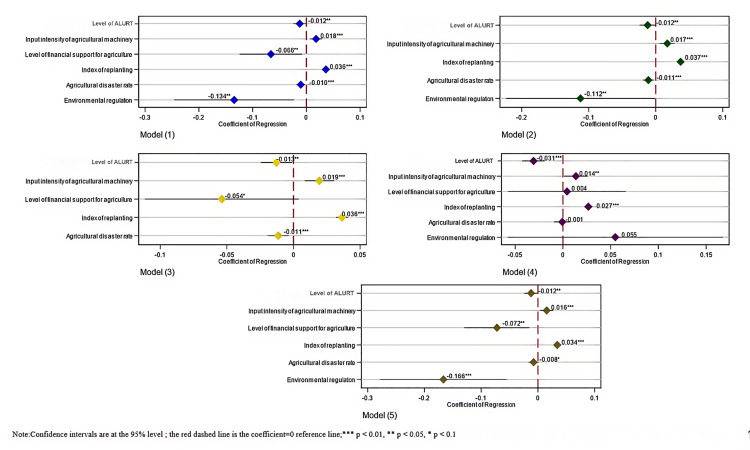
Forest plot of the impact of ALURT on CEI of croplands. (a) The five subplots in the figure represent the impact coefficients of ALURT and some columns of control variables on the CEI of cropland under different models; (b) The X-axis represents the impact coefficients and the Y-axis represents the different variables; (c) Confidence intervals are at the 95% level, the red dashed line is the coefficient = 0 reference line; (d) Asterisks labeled on the coefficient values indicate significance: *** p < 0.01, ** p < 0.05, * p < 0.1.

The influence of ALURT on the CEI from croplands was analyzed using the double fixed effect model (Eq. 1), and the findings are presented in model (1) of Fig 4. The results showed that ALURT adversely affected the CEI from croplands; specifically, an increased proportion of transferred cropland areas led to a more substantial reduction in CEI. Consequently, Hypothesis 1, which suggests that a higher level of ALURT could reduce cropland CEI, was confirmed. Among the control variables, increases in agricultural machinery inputs can significantly increase the CEI from arable land. Although agricultural mechanization is a key indicator of modern agricultural development and greatly enhances production efficiency, it requires substantial energy. The combustion of this energy leads to significant emissions of carbon dioxide and other GHG, thereby increasing cropland CEI. Increased financial support for agriculture can reduce the CEI of croplands. Enhanced investment in financial support improved farmland water conservancy facilities, optimized crop planting structures, and promoted the adoption of low-carbon agricultural technologies, thereby lowering CEI. Conversely, higher replanting index was related to increased average cropland carbon emissions. A high replanting index indicats that more crops are planted annually on the same cropland, which typically necessitates increased soil management and inputs. Consequently, the average cropland CEI increased. A higher agricultural disaster rate correlated with a lower cropland CEI. This result was attributed to the calculation method used in this study, which included carbon emissions from cropland utilization and management. Following an agricultural disaster, the subsequent utilization of cropland was affected, leading to post-disaster restoration efforts and reduced inputs compared with normal cropland applications. Environmental regulations decreased the CEI of arable lands. Environmental regulations facilitated the adoption of low-carbon technologies, encouraged adjustments in the agricultural structure, and enhanced the efficient application of agricultural waste, thereby decreasing the CEI.

#### Robustness check.

A robustness test was conducted in two main steps. The first part involved adjusting the control variables, replacing the models, and substituting the samples to verify the robustness of the primary effect analysis. The second part addressed the endogeneity issue by using instrumental variables.

Models (2) and (3) in Fig 4 show the outcomes of adjusting the control variables. The implemented replacement estimation model was an individual fixed-effects model(the results are shown in model (4) of Fig 4). The replacement samples (the results are shown in model (5) of Fig 4) involved the removal of one province’s observations. The consistently negative impact coefficients confirmed the robustness of the main effect estimation results.

To address potential endogeneity, this study utilized the two-stage least squares method to analyze the instrumental variables based on Equations (2) and (3). The results are presented in Table 4. Initially, the proportion of the outworkers’ labor force was utilized as the instrumental variable to estimate the level of ALURT, yielding the predicted ALURT degree. This fitted value was then used in the second regression to determine the unbiased impact coefficient. The first-stage results are shown in column (1) of Table 4, and the second-stage results are shown in column (2). The findings indicated a significantly negative impact of the fitted level, demonstrating that the main effect results remained robust after accounting for endogeneity.

**Table 4:** Results of endogeneity test for outgoing labor share as an instrumental variable.

	(1) Level of ALURT	(2) Intensity of carbon emissions from cropland
Percentage of labor force working outside the home	0.432*** (0.124)	
Level of ALURT		-0.225*** (0.075)
Input intensity of agricultural machinery	0.009 (0.068)	0.036** (0.018)
Level of financial support for agriculture	-2.721*** (0.304)	-0.802*** (0.198)
index of replanting	-0.044 (0.028)	0.023*** (0.006)
Agricultural disaster rate	-0.181* (0.097)	-0.043 (0.030)
environmental regulation	0.797 (0.974)	-0.196 (0.266)
constant	0.554*** (0.082)	0.191*** (0.054)
N	270	270

Standard errors in parentheses;*** p < 0.01, ** p < 0.05, * p < 0.1

As this study utilized only one instrumental variable, over-identification could not be an issue. Consequently, endogeneity testing was performed to assess whether the explanatory variables were exogenous and whether the instrumental variables were weak. The Hausman test evaluated the exogeneity of the farmland turnover level variable, whereas Durbin’s score test was used for endogeneity in the model. The statistic for Durbin’s test was large (31.564) with a small p-value (p = 0.0000), thus significantly rejecting the null hypothesis at a high statistical significance level. Similarly, the Wu-Hausman test demonstrated a large F-statistic (34.6834) and a very small p-value (p = 0.0000), which rejected the null hypothesis of exogeneity at a high statistical significance level. The weak instrumental variable and non-identifiable tests revealed an F-value of 12.045 for the first-stage regression, which exceeded the critical value of 8.96 for a 15% bias, confirming that the proportion of the labor force working outside the home was not a weak instrumental variable.

### Mediation effect test

The first step of the path analysis method was verified in the main effect analysis in the previous section and will not be repeated here. In the second step, which tested the effect of the independent variables on the mediating variables, the results showed that the coefficient of the impact of ALURT on large-scale operations was 1.357. This suggests that ALURT can indeed promote large-scale operations. However, the P value was 0.515, indicating that the effect was not significant. This finding confirms that large-scale operations do not serve as a mediating factor in the relationship between ALURT and cropland CEI, thereby overturning hypothesis 2. To further investigate the mechanism through which ALURT influences cropland CEI, the level of large-scale operations was further examined as a potential threshold variable.

### Threshold effect analysis

To determine whether a threshold effect existed in the relationship between cropland CEI and ALURT, this analysis used a large-scale operation level as the threshold variable and applied the “self-help method” with 400 sample repetitions. The results, detailed in Table 5, revealed a single threshold effect, with a threshold of 13.05 mu/person. Specifically, when the scale of operation was less than 13.05 mu/person, the impact coefficient of ALURT on cropland CEI was -0. 022. However, when the scale of operation exceeded 13.05 mu/person, the impact coefficient was -0.037. This demonstrated that as the cropland operation scale increased, the inhibition of ALURT on cropland carbon emissions was further enhanced.

**Table 5:** Results of the threshold effect analysis.

	Intensity of carbon emissions from croplands
Level of ALURT (Level of large-scale operation≤13.05)	-0.022*** (0.006)
Level of ALURT (Level of large-scale operation>13.05)	-0.037*** (0.006)
constant	0.021*** (0.006)
Control variable	control
N	270
R2	0.458

The standard errors are presented in parentheses. *** p < 0.01, ** p < 0.05, and * p < 0.1.

## Discussion

As outlined in the introduction, this study measured the CEI of croplands using the area of cultivated land, rather than the agricultural output or agricultural output value, as the denominator, and investigated the impact of ALURT on cropland CEI. The conclusions of this study are generally consistent with existing research. However, during exploration of the underlying mechanisms, it was found that the scale of operation acted as a threshold variable rather than a mediating factor in the relationship between ALURT and cropland CEI. This finding differs from those of previous [38] and may be attributed to variations in the study’s time period and indicator selection. To effectively promote ALURT and reduce cropland CEI, efforts should focus on enhancing the level of large-scale operations to achieve more efficient agricultural production and lower CEI. However, owing to limitations in the research data, the maximum operation scale in the sample was 41.571 mu/person, leaving the effects of operation scales beyond this threshold unexplored. Further research is required to address this gap by expanding the sample range or adjusting the sample indicators.

## Conclusions

Based on panel data from 30 provinces in China from 2014 to 2022, this study assessed the impact of ALURT on the CEI of arable land and its underlying mechanisms. The following conclusions were drawn from this study. (1) Both the arable land CEI and total carbon emissions in China peaked in 2015 and have since gradually declined. At the provincial level, the CEI of arable land in most provinces fluctuated within a narrow range, showing no clear upward or downward trend. However, four provinces (Fujian, Zhejiang, Hainan, and Guangdong) recorded the highest CEI values, which also exhibited significant fluctuations with notable increases between 2018 and 2019. (2) ALURT significantly reduced the CEI of cultivated land. (3) Regarding the effect of ALURT on the CEI of cropland, the scale of farmland operation did not act as a mediating factor but rather as a moderating variable. The relationship between CEI and ALURT was nonlinear. Specifically, the inhibition of carbon emissions strengthened when the scale of operation exceeded 13.05 mu/person.

## References

[1] Sheng M, Shi W, Lin X, Wu B, Wu S. Revealing the spatiotemporal evolution pattern and convergence law of agricultural land transfer in China. PLoS ONE. 2024;19(6):e0300765. doi: 10.1371/journal.pone.0300765 38843132 PMC11156318

[2] Li J, Jiang L, Zhang S. How Land transfer affects agricultural carbon emissions: evidence from China. Land. 2024;13(9):1358. doi: 10.3390/land13091358

[3] Ma G, Lv D, Jiang T, Luo Y. Can land transfer promote agricultural green transformation? The empirical evidence from China. Sustainability-Basel. 2023;15(18):13570. doi: 10.3390/su151813570

[4] Rong J, Hong J, Guo Q, Fang Z, Chen S. Path mechanism and spatial spillover effect of green technology innovation on agricultural CO2 emission intensity: a case study in Jiangsu Province, China. Ecol Indic. 2023;157:111147. doi: 10.1016/j.ecolind.2023.111147

[5] Guo Z, Zhang X. Carbon reduction effect of agricultural green production technology: a new evidence from China. Sci Total Environ. 2023;874:162483. doi: 10.1016/j.scitotenv.2023.162483 PMID:36858221

[6] Grewer U, Nash J, Gurwick N, Bockel L, Galford G, Richards M, et al. Analyzing the greenhouse gas impact potential of smallholder development actions across a global food security program. Environ Res Lett. 2018;13(4):044003. doi: 10.1088/1748-9326/aab0b0

[7] Bauernschuster S, Pichler M, Nanhthavong V, Bernhard R, Epprecht M, Gingrich S. Carbon emissions from land acquisitions in Laos. Ecol Soc. 2022;27(3). doi: 10.5751/es-13395-270345 PubMed PMID: WOS:000894210200004.

[8] Jiang Y, Qian H, Huang S, Zhang X, Wang L, Zhang L, et al. Acclimation of methane emissions from rice paddy fields to straw addition. Sci Adv. 2019;5(1):eaau9038. doi: 10.1126/sciadv.aau9038 PMID:30746466 PMC6357747

[9] Wang J, Chang G, Liu H, Yin Z, Liu P, Zhao Y, et al. Carbon balance analysis of agricultural production systems in oasis areas. Sci Rep. 2024;14(1):16698. doi: 10.1038/s41598-024-66972-4 39030311 PMC11271539

[10] Li S, Wang Z. The effects of agricultural technology progress on agricultural carbon emission and carbon sink in China. Agriculture-Basel. 2023;13(4):793. doi: 10.3390/agriculture13040793 PubMed PMID: WOS:000979514800001

[11] Wu H, Huang H, Chen W, Meng Y. Estimation and spatiotemporal analysis of the carbon-emission efficiency of crop production in China. J Clean Prod. 2022;371:133516. doi: 10.1016/j.jclepro.2022.133516

[12] Wen S, Hu Y, Liu H. Measurement and spatial–temporal characteristics of agricultural carbon emission in China: an internal structural perspective. Agriculture-Basel. 2022;12(11):1749. doi: 10.3390/agriculture12111749

[13] Zheng X, Tan H, Liao W. Spatiotemporal evolution of factors affecting agricultural carbon emissions: empirical evidence from 31 Chinese provinces. Environ Dev Sustain. 2024. doi: 10.1007/s10668-023-04337-z

[14] You Y, Tian H, Pan S, Shi H, Lu C, Batchelor WD, et al. Net greenhouse gas balance in U.S. croplands: how can soils be part of the climate solution? Global Change Biol. 2024;30(1):e17109. doi: 10.1111/gcb.17109 38273550

[15] Xia Y, Fu C, Wu H, Wu H, Zhang H, Liao A, et al. Exploring the effects of extreme weather events on methane emissions from croplands: a study combining site and global modeling. Agric Forest Meteorol. 2023;335:109454. doi: 10.1016/j.agrformet.2023.109454

[16] Shakoor A, Arif MS, Shahzad SM, Farooq TH, Ashraf F, Altaf MM, et al. Does biochar accelerate the mitigation of greenhouse gaseous emissions from agricultural soil? - A global meta-analysis. Environ Res. 2021;202:111789. doi: 10.1016/j.envres.2021.111789 34333013

[17] Wu H, Sipiläinen T, He Y, Huang H, Luo L, Chen W, et al. Performance of cropland low-carbon use in China: measurement, spatiotemporal characteristics, and driving factors. Sci Total Environ. 2021;800:149552. doi: 10.1016/j.scitotenv.2021.149552 34391149

[18] Some S, Roy J, Ghose A. Non-CO2 emission from cropland based agricultural activities in India: a decomposition analysis and policy link. J Clean Prod. 2019;225:637–46. doi: 10.1016/j.jclepro.2019.04.017

[19] Xia Y, Guo H, Xu S, Pan C. Environmental regulations and agricultural carbon emissions efficiency: evidence from rural China. Heliyon. 2024;10(4):e25677. doi: 10.1016/j.heliyon.2024.e25677 38370207 PMC10869864

[20] Chen M-Q, Zhong T-Y, Zhou B-J, Huang H-S, He W-J. Empirical research on farm households’ attitude and behaviour for cultivated land transferring and it’s influencing factors in China. Agr Econ - Czech. 2010;56(9):409–20. doi: 10.17221/93/2009-agricecon

[21] Wang J, Xin L, Wang Y. How farmers’ non-agricultural employment affects rural land circulation in China? J Geogr Sci. 2020;30(3):378–400. doi: 10.1007/s11442-020-1733-8

[22] He X, Liu W. Analysis of spatial patterns and influencing factors of farmland transfer in China based on ESDA-GeoDetector. Sci Rep. 2024;14(1):12485. doi: 10.1038/s41598-024-62931-1 38816491 PMC11139945

[23] Liu C, Wang Y, Yang B, Zheng L. How do socioeconomic differentiation and rural governance affect households’ land transfer decisions? Evidence from China. Land Degrad Dev. 2024;35(2):884–97. doi: 10.1002/ldr.4959

[24] Udimal TB, Liu E, Luo M, Li Y. Examining the effect of land transfer on landlords’ income in China: An application of the endogenous switching model. Heliyon. 2020;6(10):e05071. doi: 10.1016/j.heliyon.2020.e05071 33033761 PMC7533368

[25] Zang D, Yang S, Li F. The relationship between land transfer and agricultural green production: a collaborative test based on theory and data. Agriculture-Basel. 2022;12(11):1824. doi: 10.3390/agriculture12111824

[26] Huo C, Chen L. The impact of land transfer policy on sustainable agricultural development in China. Sci Rep. 2024;14(1):7064. doi: 10.1038/s41598-024-57284-8 38528076 PMC10963742

[27] Wang RR, Zhang Y, Zou CM. How does agricultural specialization affect carbon emissions in China? J Clean Prod. 2022;370:133463. doi: 10.1016/j.jclepro.2022.133463

[28] Wan JJ, Jing YS, Ji MY, Sun QN. Research progress on the application of DNDC model in water and fertilizer management in paddy fields. Chinese J Ecol. 2024;43(07):2198–207. doi: 10.13292/j.1000-4890.202407.012

[29] Deininger K, Jin S. The potential of land rental markets in the process of economic development: evidence from China. J Dev Econ. 2004;78(1):241–70. doi: 10.1016/j.jdeveco.2004.08.002

[30] Dhillon R, Moncur Q. Small-scale farming: a review of challenges and potential opportunities offered by technological advancements. Sustainability. 2023;15(21):15478. doi: 10.3390/su152115478

[31] Geng N, Wang M, Liu Z. Farmland transfer, scale management and economies of scale assessment: evidence from the main grain-producing Shandong Province in China. Sustainability. 2022;14(22):15229. doi: 10.3390/su142215229

[32] Li B, Zhang JB, Li HP. Decomposition of spatial and temporal characteristics and influencing factors of agricultural carbon emissions in China. Chin J Popul Resour Environ. 2011; 21(08):80–6.

[33] Li S, Wang Z. Time, spatial and component characteristics of agricultural carbon emissions of China. Agriculture. 2023;13(1):214. doi: 10.3390/agriculture13010214

[34] Wu FL, Li L, Zhang HL, Chen F. Effects of conservation tillage on net carbon release from agro-ecosystems. Chin J Ecol. 2007;(12):2035–9.

[35] Dubey A, Lal R. Carbon footprint and sustainability of agricultural production systems in Punjab, India, and Ohio, USA. J Crop Improv. 2009;23(4):332–50. doi: 10.1080/15427520902969906

[36] West T O. Marland G. A synthesis of carbon sequestration,carbon emissions,and net carbon flux in agriculture:comparing tillage practices in the United States. Agric Ecosyst Environ. 2002;91(1-3):217–32. doi: 10.1016/S0167-8809(01)00233-X

[37] Zhi J, Gao JX. Comparative analysis of carbon emissions from food consumption by urban and rural residents in China. Prog Geogr. 2009; 28(03):429–34.

[38] Song H, Jiang H, Zhang S, Luan J. Land circulation, scale operation, and agricultural carbon reduction efficiency: evidence from China. Discrete Dyn Nat Soc. 2021;2021:1–12. doi: 10.1155/2021/9288895

